# Editorial: Engineered Targeted Cancer Immunotherapies

**DOI:** 10.3389/fonc.2022.953175

**Published:** 2022-07-04

**Authors:** Massimo Fantini, Roberto Bei

**Affiliations:** ^1^ Precision Biologics, Inc., Bethesda, MD, United States; ^2^ Department of Clinical Sciences and Translational Medicine, University of Rome “Tor Vergata”, Rome, Italy

**Keywords:** cancer immunotherapy, monoclonal antibodies, CAR-T cells, CAR-NK cells, antibody-dependent cell-mediated cytotoxicity

Conventional cancer therapies, including surgery, radiotherapy and chemotherapy showed good effects in the treatment of patients with early-stage cancers, but they often fail to cure many patients that develop metastasis in different organs.

To overcome this issue more selective therapies, such as immunotherapy, have been developed in the last few decades.

The aim of immunotherapy is to enhance the power of immune system to target cancer, leading to a selective killing of cancer cells and a concomitant preservation of normal tissues.

Unfortunately, cancer cells use several mechanisms to impair the efficacy of immunotherapy, such as expression of neo-antigens, over-expression of immunosuppressive molecules (IDO, PD-L1), accumulation of myeloid-derived suppressor cells (MDSCs) and regulatory T cells in the tumor microenvironment (TME).

To improve immunotherapy efficacy and to overcome the inhibitory activity of the TME on the immune system, engineered targeted cancer immunotherapies have been developed. These include bispecific monoclonal antibodies, immunotoxins, fusion proteins, chimeric antigen receptor (CAR)-T cells, gene therapy and monoclonal antibodies (mAbs) with antibody-dependent cell-mediated cytotoxicity (ADCC) or complement dependent cytotoxicity (CDC) activity.

CAR-T cell technology is based on the isolation of patient’s T lymphocytes, which are then engineered to express chimeric antigen receptors (CARs). The modified T lymphocytes can recognize and kill cancer cells in a manner that does not involve the major histocompatibility complex (MHC). After proliferation *in vitro*, CAR-T cells are reinfused into the patient (Lin et al.).

CAR-T cells achieved promising results as immunotherapy, especially against hematological malignancies, where they showed impressive response with high target specificity.

In this regard, in the review from Gambella et al. is reported that CAR-T cells targeting CD19 showed promising results in the treatment of diffuse large B-cell lymphoma (Gambella et al.).

In their original research, Wang et al. observed that Bryostatin, a member of a family of cyclic polyketides, which interacts with the diacylglycerol biding site of the C-1 regulatory domain of protein kinase C, activates CAR T-cell antigen-non-specific killing (CTAK), and CAR-T NK-like killing for Pre-B acute lymphocytic leukemia (ALL) through the modulation of both CD19 and CD22 expression on leukemia cells. This modulation allows for a greater degree of CAR-mediated leukemia cell killing. (Wang et al.).

However, in patients with solid tumors, CAR-T cell therapy did not achieve yet a good objective response and this phenomenon is due to the ability of TME of solid tumors to inactivate CAR-T cells.

All existing CAR-T cells available on the market are autologous (made with same patient-derived T lymphocytes) to avoid severe alloimmune rejection due to a mismatch of MHC between the donor and the recipient.

As explained in the review from Lin et al., to improve the efficacy of CAR-T cells, costimulatory molecules, such as CD28 or 4-1BB, were incorporated into CAR structure to promote CAR-T cells survival and functionality *in vivo* (second and third generation CAR). In addition, CAR-T cells have been further engineered to secrete cytokines (fourth generation CAR) which allow to CAR-T to be more viable and to activates other immune cells (Lin et al.).

In their review, Zhang et al. showed the importance to use gene-edited interleukin CAR-T cells therapy as a novel strategy for the treatment of malignancies. The most used cytokines used to construct fourth generation CAR are interleukins including IL-7, IL-12, IL-15, IL-18, IL-21 and IL-23. These CAR-T cells include co-expression of single interleukin, two interleukins, interleukin combined with other cytokines, interleukin receptors, interleukin subunits, and fusion inverted cytokine receptors (ICR). There are several Phase I and Phase I/II clinical trials evaluating the safety and efficacy of gene-edited interleukin-CAR-T (fourth generation CAR), involving hematological tumors and solid tumors (Zhang et al.). Another efficient gene editing process to improve efficacy of CAR-T cells is the CRISPR/Cas9 strategy for the editing of human primary NK and T Cells as reported by Elmas et al. For example, CRISPR/Cas9 has been used to knock down TGF-β receptor II (TGFBR2) to reduce CAR-T cells exhaustion and to enhance CAR-T cells anti-tumor activity. In addition, CRISPR/cas9 knock down of granulocyte-macrophage colony-stimulating factor (GM-CSF) was useful to decrease cytokine release syndrome (CRS) and neuroinflammation linked to CAR-T cell therapy (Elmas et al.).

Despite these improvements, there are still some safety concerns on the use of autologous CAR-T cells, including CRS and neurotoxicity caused by CAR-T cells overactivation. In addition, autologous CAR-T cells have high cost and intensive manufacturing process, which slow down their quick availability for the patient.

Lin et al. in their review explained that one strategy to further improve CAR-T cells safety and efficacy is to employ universal CAR-T (UCAR-T) cell therapy, which consist of allogeneic CAR-T cells that are taken from healthy donors. UCAR-T cells share the same engineering process and mechanisms of action of autologous CAR-T, but are cheaper than autologous CAR-T, have a much less intensive manufacturing process, can be immediately available to cancer patients and showed promising results in treating T-cells malignancies. To reduce the Graft-Versus-Host Disease (GvHD) and rejection, UCAR-T cells underwent to additional gene editing processes, such as knock out of the TCR, genetic ablation of MHC-I and/or MHC-II and editing of CD7 to prevent the fratricide in CD7 UCAR-T cells (Lin et al.).

A more recent and promising approach is the employment of chimeric antigen receptor-engineered NK (CAR-NK) cells. In their review, Baysal et al. reported that CAR-NK can be obtained either through lenti-/retroviral transduction of primary adult natural killer (NK) cells or through the engineered immortalized NK-92 cells. CAR-NK cells have several advantages over CAR-T cells, including more robustness, reduction of frequency of cytokine release syndrome, suppression of GvHD induced by CAR-T cells (Baysal et al.). In this regard, in recent years, several clinical trials have investigated the use of CAR-NK cells as therapeutic approach against hematological malignancies and indicated the possibility of adopting CAR-NK therapy for patients with high-risk B cell lymphoma and leukemia. CAR-NK cells can also be equipped with on-board cytokines, such as IL-15, to enhance both persistence and cytotoxicity against tumor cells (Gambella et al.).

Beyond T cells and NK cells, also macrophages can be engineered to improve cancer immunotherapy.

In the review from Ding et al. are reported different methods to create engineered macrophages for cancer therapy *via* nanotechnology and genetic manipulation. Since macrophages have a great ability to infiltrate tumors, a promising strategy to deliver anti-cancer drugs in the TME is to load macrophages with nanoparticles (NPs). NPs can deliver a variety of anticancer agents, such as chemotherapeutic drugs, targeted drugs, messenger RNA, small interfering RNA, and the CRISPR/Cas9 genetic editing system, and many studies have demonstrated that NP-loaded macrophages (NPL-Ms) can deliver the anti-cancer drug in a more efficient manner to tumor cells, leading to a strong antitumor effect. In addition, the review from Ding et al. showed also that macrophages engineered to express CARs can efficiently migrate to tumor sites and to kill tumor cells through phagocytosis. After reaching TME, these engineered macrophages can significantly subvert TME immunosuppressive activity and, in turn, enhance T cell-mediated anticancer immune responses (Ding et al.).

Another strategy to improve immunotherapy is to engineer mAbs targeting tumor antigens.

Important targets of anti-cancer mAbs are pathways mediated by the epidermal growth factor receptor (EGFR), CD20, vascular endothelial growth factor (VEGF), and the programmed cell death protein-1 (PD-1)/programmed cell death protein-1 ligand (PD-L1).

Although the immunotherapy with mAbs has increased survival of cancer patients, the lack of tumor antigens, uncontrolled activation of oncogenes, increased activity of regulatory T cells and MDSCs in the TME can lead to the resistance to immune checkpoints inhibitors (ICIs)-based therapy and to its subsequent failure.

To overcome this issue, mAbs were engineered to have different mechanisms of action. In this regard, mAbs able to mediate ADCC may contribute to improve the clinical response of cancer patients treated with ICIs.

Examples of clinically approved mAbs that can mediate ADCC include trastuzumab, rituximab, cetuximab, avelumab.

Baysal et al. reported that one interesting strategy to potentiate the ADCC activity mediated by mAbs is the employment of adoptive NK cells to restore NK cell functionality of cancer patients that is often impaired by immunosuppressive activity of TME. Authors suggested that a promising approach, in evaluation in different clinical trials, is the combination between cetuximab, which targets the epidermal growth factor receptor (EGFR) expressed in breast, lung, colorectal, head and neck cancers, and adoptive transfer of autologous or allogenic expanded NK cells (Baysal et al.).

The employment of allogenic expanded NK cells has the advantage of being a good alternative to autologous NK cells due to the limited number of patient-derived NK cells. Other benefits of allogenic NK cells include the possibility to obtain NK cells from healthy donors and the ability to produce high quantity of engineered NK cell lines with a greater antitumor activity.

Baysal et al. also reported that the anti-tumor activity of allogenic NK cells in combination with cetuximab can be enhanced by stimulation of NK cells with cytokines such as IL-2, IL-12, IL-15, IL-21. Stimulation of NK cells with these cytokines leads to enhancement of the antitumor effects of NK cells against various tumor types and significantly increases cytokine and chemokine secretions which, in turn, stimulate the infiltration of CD8+ T cells into the tumor. Several clinical trials showed promising clinical responses and a tolerable safety profile using cetuximab in combination with NK stimulated with these cytokines in different cancer types (Baysal et al.).

In their original research article, Klewinghaus et al. suggested that another efficient strategy to kill EGFR^+^ cells could be the employment of cattle-derived ultralong CDR-H3 common light chain bispecific antibodies targeting EGFR on tumor cells as well as natural cytotoxicity receptor NKp30 on NK cells. These engineered bispecific antibodies elicited potent NK cell killing of EGFR-overexpressing tumor cells as well as robust release of proinflammatory cytokine interferon-γ (IFN- γ) *in vitro*. Since IFN-γ can inhibit suppressive immune cell subsets and redirect NK, NKT and T cell trafficking into tumors, the stimulation of NK cells to release IFN-γ by these types of bispecific antibodies might be a promising strategy to improve antibody-based immunotherapy in clinic (Klewinghaus et al.).

Chasov et al. reported promising new humoral and cell-based immunotherapies for targeting p53 mutant cancers.

Authors showed that the peptide neoantigens from a proteolytically processed mutant p53 protein are presented by APCs to B and T cells to activate the immune response. To this end, an interesting approach is based on bispecific TCRm antibodies that bind to both TCR and the peptide on MHC (pMHC) presenting the mutant p53 antigen. The scope of this approach is to enhance the presentation to T cells of mutant p53 peptides to stimulate T cells to destroy cancer cells bearing mutant p53 without affecting the normal cells with wild type p53 (Chasov et al.).

Kooti et al. reported studies showing that oncolytic viruses (OVs) can represent a valid alternative to CARs and engineered mAbs to kill cancer cells.

Oncolytic viruses (OVs) include a group of viruses that selectively recognize and kill malignant cells, without affecting the surrounding health cells. OVs can kill cancer cells through several mechanisms, including direct cytotoxicity, induction of immune-mediated cytotoxicity and disruption of tumor vasculature.

In addition, OVs can favor recruitment of immune cells, such as cytotoxic T lymphocytes, dendritic cells, NK cells and phagocytic cells in the TME to induce immune cell death of cancer cells. To improve their efficacy OVs are often engineered to express immune-stimulatory (IL-2, IL-4, IL-12 and GM-CSF) and pro-apoptotic (tumor necrosis factor alpha, p53 and TRAIL) genes (Kooti et al.).

The treatment of hepatocellular carcinoma (HCC), one of the most common malignancies globally, and multiple myeloma (MM), is benefiting from some of engineered cancer immunotherapies mentioned above. In the review from Miao et al. is reported how ICIs (anti PD-1/PD-L1 and cytotoxic T-lymphocyte antigen-4 (CTLA-4) mAbs, alone or in combination), tumor vaccines, engineered NK cells, CAR-T cells are widely used in clinic and in clinical trials for the treatment of HCC (Miao et al.).

Similarly, the review from Guo et al. showed that, for thetreatment of MM, the most promising engineered cancer immunotherapies evaluated in clinical trials are antibody-drug conjugates (ADCs), second-generation CAR-T cells and CAR-NK cells (Guo et al.).

The big challenge now is to evaluate the combination of engineered targeted cancer immunotherapies with conventional treatment methods to evaluate if this strategy can produce synergistic effects and a better efficacy for the treatment of blood and solid tumors.

## Author Contributions

All authors listed have made a direct and intellectual contribution to the work and approved the submitted version.

## Conflict of Interest

MF is an employee of Precision Biologics, Inc., a biotech company producing monoclonal antibodies for the treatment of solid tumors (Phase 1/2 clinical trials). MF holds a patent for one of these monoclonal antibodies.

The remaining author declare that the research was conducted in the absence of any commercial or financial relationships that could be construed as a potential conflict of interest.

## Publisher’s Note

All claims expressed in this article are solely those of the authors and do not necessarily represent those of their affiliated organizations, or those of the publisher, the editors and the reviewers. Any product that may be evaluated in this article, or claim that may be made by its manufacturer, is not guaranteed or endorsed by the publisher.

